# The Varied Presentation of Appendiceal Mucinous Neoplasms: A Rural Case Series

**DOI:** 10.7759/cureus.104575

**Published:** 2026-03-02

**Authors:** David Vu, Renata Pajtak, Paul Strauss

**Affiliations:** 1 General Surgery, Austin Health, Melbourne, AUS; 2 General Surgery, Central Gippsland Health, Sale, AUS

**Keywords:** appendiceal mucinous neoplasms, cytoreductive surgery, hyperthermic intraperitoneal chemotherapy, low-grade appendiceal mucinous neoplasm, peritoneal neoplasms, pseudomyxoma peritonei

## Abstract

Appendiceal mucinous neoplasms (AMNs) are a rare and heterogeneous disease. Failure to promptly recognize AMNs can lead to rupture, peritoneal seeding, and pseudomyxoma peritonei (PMP). We present five cases in rural Australia in whom AMN was suspected or identified preoperatively, intraoperatively, or via histopathology. Three of our cases were males, with an age range of 47-89 years. Intraoperative findings varied from dilated appendices with mucinous content, large well-circumscribed masses, to perforated tumors with peritoneal dissemination and PMP. Surgical interventions included laparoscopic appendicectomies, open appendicectomy via Lanz incision or midline laparotomy, and a diagnostic laparotomy for biopsy of a perforated tumor. One patient required cytoreductive surgery and hyperthermic intraperitoneal chemotherapy, and another received palliative chemotherapy. In conclusion, familiarity with AMN treatment is pertinent to their rising incidence - we are the first to explore strategies for management of this rare but important condition in rural Australia.

## Introduction

Appendiceal mucinous neoplasms (AMNs) are a rare and heterogeneous disease, though incidence has increased by 415% and mortality by 130% [1]. The literature defines three different histological entities: low‑grade AMN (LAMN), high‑grade AMN (HAMN), and mucinous adenocarcinoma [2]. LAMNs are low-grade, well-differentiated tumors; however, upon rupture, they can produce widespread intraperitoneal deposits called pseudomyxoma peritonei (PMP), resulting in high morbidity and mortality [2]. These lesions necessitate cytoreductive surgery (CRS) and hyperthermic intraperitoneal chemotherapy (HIPEC) [2].

Though accounting for only 0.2%-0.3% of cases receiving appendicectomy [3], recent literature suggests that incidence may be higher in adult rural cohorts, with rates as high as 7% [4]. As such, these rural centers are met with the challenge of managing a rare pathology without the multidisciplinary cancer resources available to tertiary centers [5,6]. This case series thereby examines five cases in rural Australia to identify the wide spectrum of AMN presentations, management principles, and strategies to deliver gold-standard care in a rural environment.

This article was previously presented as an oral presentation at the Royal Australasian College of Surgeons Annual Scientific Congress 2025 on November 15, 2025.

Methodology

A retrospective analysis was performed on five patients aged 47-89 years presenting to Central Gippsland Health (CGHS) between 2014 and 2023 (Monash modified category 4). Collected data included gender, age, blood tests, diagnostic imaging, operative intervention, and histopathology via de-identified patient medical records. 

This study did not require ethical approval. Data access was approved by the Board of Directors at CGHS hospital. Permission for publication was obtained in accordance with the 1964 Declaration of Helsinki, its subsequent amendments, and comparable ethical standards. Informed consent was obtained from each patient for the publication of this study and the accompanying images.

## Case presentation

Case 1

A 72-year-old male presented to the emergency department (ED) with two weeks of worsening right upper abdominal pain migrating to the right iliac fossa (RIF). He had a history of atrial fibrillation and hypertension. On examination, he had rebound tenderness in the RIF. His pathology demonstrated normal hemoglobin (Hb), white cell count (WCC), and C-reactive protein (CRP), with high cancer antigen 19-9 (CA 19-9) and carcinoembryonic antigen (CEA), though cancer antigen 125 (CA 125) was normal (Table 1).

**Table 1:** Summary of laboratory results (Case 1). Hb, hemoglobin; WCC, white cell count; CRP, C-reactive protein; CEA, carcinoembryonic antigen; CA 19-9, cancer antigen 19-9; CA 125, cancer antigen 125

Laboratory result	Normal reference range	Results (Case 1)
Hb	135-175 g/L (male), 115-165 g/L (female)	146
WCC	4.0-11 x 10^9^/L	7.4
CRP	0-3 mg/L	2
CEA	0-4.9 µg/L	63
CA 19-9	0-36.0 U/mL	1,930
CA 125	0-34 U/mL	29

**Figure 1:**
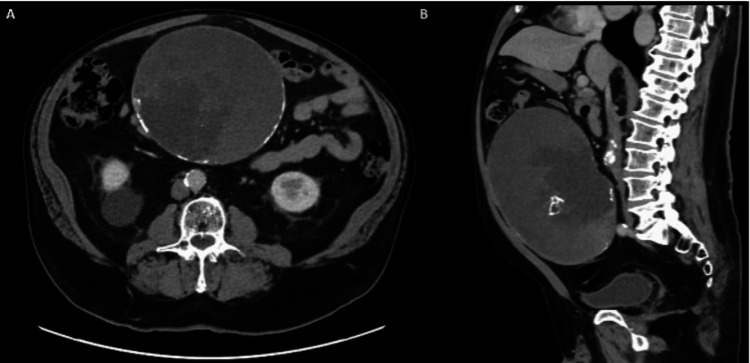
Computed tomography of the abdomen and pelvis with contrast (Case 1). (A) Sagittal view and (B) axial view demonstrating a 16.6 x 18 x 20.9 cm, partly calcified, circumscribed heterogeneous intraperitoneal mass later found to be an appendiceal mucinous neoplasm.

**Figure 2:**
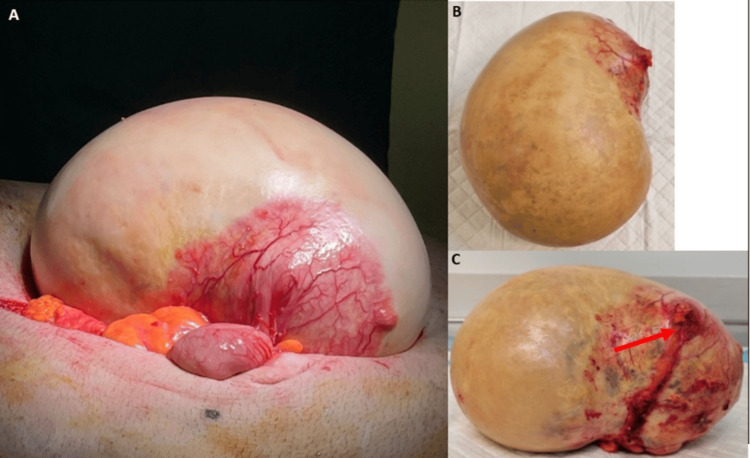
Large appendiceal mass accessed via midline laparotomy (Case 1). (A) Overhead view of the mass attached to the appendix; (B) overhead view after excision; (C) view showing the prior appendiceal attachment site, indicated by the red arrow.

He underwent computed tomography of the abdomen and pelvis (CTAP), which demonstrated a large RIF mass (Figure 1). He was discharged home with a plan for a multidisciplinary meeting (MDT), which recommended explorative laparotomy. Two months later, the patient underwent a midline laparotomy, which identified a large, well-circumscribed appendiceal mass (Figure 2). His postoperative course was uncomplicated, and he was discharged after six days.

Histopathology revealed adenocarcinoma in situ of the appendix with mucocele transformation. The mucocele had a fibromuscular wall that was not invaded by tumor, though it did extend luminally. Postoperatively, the patient underwent positron emission tomography-CT (PET-CT), which reported no locoregional, nodal, or distant metastatic disease. He was followed up locally at six-month intervals and remains disease-free after three years of follow-up.

Case 2

An 89-year-old male presented to the ED with four days of right-sided abdominal pain, inability to pass flatus, and constipation. He had a history of type 2 diabetes on insulin, recurrent deep vein thrombosis on warfarin, bronchiectasis, and osteoarthritis. His pathology showed high WCC and CRP, with borderline CEA levels (Table 2).

**Table 2:** Summary of laboratory results, with trend of inflammatory markers two days after presentation (Case 2). Hb, hemoglobin; WCC, white cell count; CRP, C-reactive protein; CEA, carcinoembryonic antigen; CA 19-9, cancer antigen 19-9; CA 125, cancer antigen 125

Laboratory result	Normal reference range	Results (Case 2, on presentation)	Results (Case 2, two days after presentation)
Hb	135-175 g/L (male), 115-165 g/L (female)	N/A	125
WCC	4.0-11 x 10^9^/L	15.2	19.9
CRP	0-3 mg/L	394	380
CEA	0-4.9 µg/L	5.1	n/A
CA 19-9	0-36.0 U/mL	N/A	N/A
CA 125	0-34 U/mL	N/A	N/A

**Figure 3:**
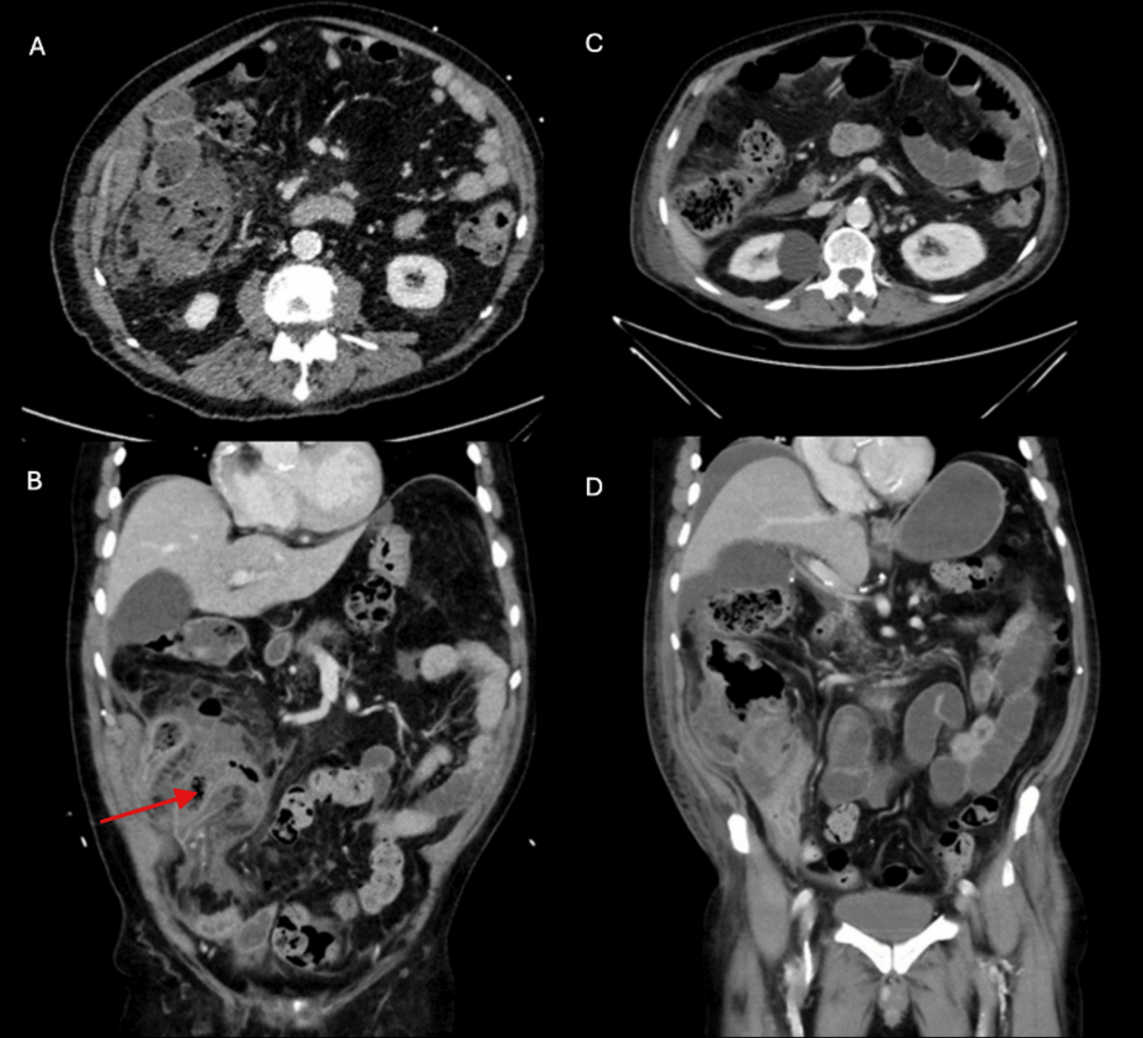
Computed tomography of the abdomen and pelvis with contrast (Case 2). (A) axial section and (B) coronal section on initial presentation, with the red arrow pointing to a markedly thickened appendix measuring up to 25 mm, with extensive inflammatory changes, adjacent fluid densities, and free fluid in the pelvis. (C) Axial section and (D) coronal section of a CTAP performed two days later, demonstrating a collection lateral to the caecum that had perforated, with early signs of small bowel obstruction. There was a suggestion of an underlying caecal malignancy that had perforated. CTAP, computed tomography of the abdomen and pelvis

CTAP on presentation demonstrated a dilated appendix (Figures 3A-3B), and he was admitted for conservative management with piperacillin-tazobactam. Due to stagnant inflammatory markers (WCC 19.9, CRP 380), he was rescanned two days later, demonstrating appendiceal rupture (Figures 3C-3D). These results prompted the decision to proceed with diagnostic laparoscopy - this revealed an inflammatory mass incorporating the terminal ileum, adjacent small bowel loops, and caecum. There were mucus deposits in the pelvis, left lower quadrant, and right upper quadrant. Multiple biopsies were taken from the mass, and free fluid was sent for analysis.

His postoperative course was complicated by ileus, managed with a nasogastric tube and total parenteral nutrition. He was discharged 24 days postoperatively. Histopathology showed metastatic adenocarcinoma with mucinous/signet ring cell features, with cytology suggesting a colorectal origin. PET-CT demonstrated extensive peritoneal disease. After the MDT discussion, he was referred to a tertiary cancer center for oncology input. Due to age and widely disseminated peritoneal disease, he was deemed a non-operative candidate, commenced palliative chemotherapy locally, and passed away 133 days postoperatively.

Case 3

**Figure 4:**
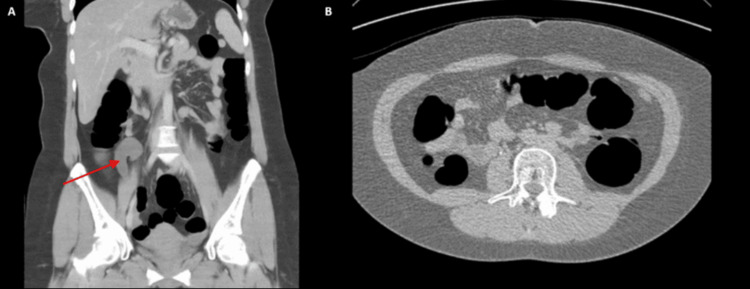
Computed tomography colonography (Case 3). (A) Coronal section with the red arrow indicating an apparent mucocele of the distal two-thirds of the appendix, measuring approximately 8 cm in length and 2.5 cm in diameter; (B) axial section.

A 47-year-old female presented to the ED with a one-week history of right-sided abdominal pain and diarrhea. She had a history of pyelonephritis, endometriosis, hypertension, gastroesophageal reflux, and a duodenal ulcer. Pathology demonstrated raised CRP (Table 3). On examination, she had RIF tenderness. To investigate these symptoms, she underwent an outpatient CT colonography, which demonstrated a suspected AMN (Figure 4).

**Table 3:** Summary of laboratory results (Case 3). Hb, hemoglobin; WCC, white cell count; CRP, C-reactive protein; CEA, carcinoembryonic antigen; CA 19-9, cancer antigen 19-9; CA 125, cancer antigen 125

Laboratory result	Normal reference range	Results (Case 3)
Hb	135-175 g/L (male), 115-165 g/L (female)	137
WCC	4.0-11 x 10^9^/L	8.2
CRP	0-3 mg/L	239
CEA	0-4.9 µg/L	N/A
CA 19-9	0-36.0 U/mL	N/A
CA 125	0-34 U/mL	N/A

She was admitted for a laparoscopic appendicectomy, which revealed a dilated, inflamed appendix. Her postoperative course was uncomplicated. Histopathology demonstrated a mucinous adenoma with clear margins and no extra-appendiceal spread. She completed five years of local surveillance at six-month intervals without recurrence.

Case 4

**Figure 5:**
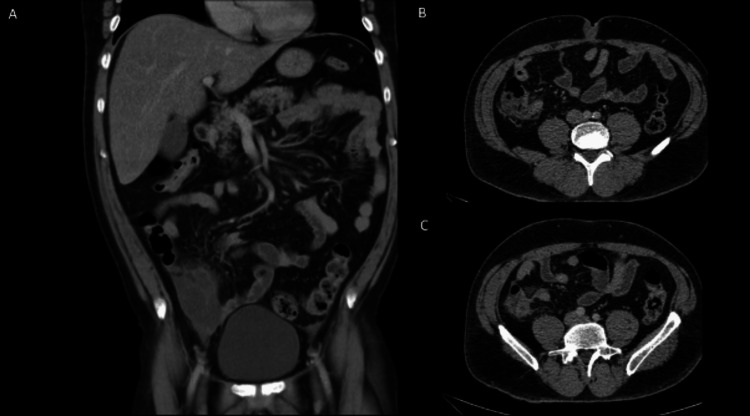
Computed tomography of the abdomen and pelvis with contrast (Case 4). (A) Coronal section; (B) axial section demonstrating a 7 cm diameter fluid collection in the right iliac fossa, lateral and inferior to the cecum. Centrally within this fluid collection, there was a low-grade, tubular, grossly thick-walled structure suggestive of a large appendiceal abscess.

A 53-year-old male presented to the ED with a one-week history of RIF pain, which had worsened in the last day with radiation to his right testicle. He had a history of type 2 diabetes on insulin, irritable bowel syndrome, and hypercholesterolemia. On examination, he had RIF tenderness with an associated palpable swelling above the cord on the right testicle. Pathology revealed raised WCC and CRP (Table 4). CTAP demonstrated a fluid collection suggestive of an appendiceal abscess (Figure 5). 

**Table 4:** Summary of laboratory results (Case 4). Hb, hemoglobin; WCC, white cell count; CRP, C-reactive protein; CEA, carcinoembryonic antigen; CA 19-9, cancer antigen 19-9; CA 125, cancer antigen 125

Laboratory result	Normal reference range	Results (Case 4)
Hb	135-175 g/L (male), 115-165 g/L (female)	140
WCC	4.0-11 x 10^9^/L	13
CRP	0-3 mg/L	38
CEA	0-4.9 µg/L	N/A
CA 19-9	0-36.0 U/mL	N/A
CA 125	0-34 U/mL	N/A

The patient was admitted for an open appendicectomy via a Lanz incision. Intraoperatively, a dilated, mucinous tumor that had ruptured into the periappendiceal and pelvic area was identified. Histopathology demonstrated a mucinous neoplasm of uncertain malignant potential/LAMN with cellular mucin present at the resection margin. The patient's postoperative course was uncomplicated, and was discharged after three days. After MDT discussion, the patient was referred to a tertiary center for CRS/HIPEC due to the presence of PMP, where they received right hemicolectomy, omentectomy, and cholecystectomy. The patient was discharged on postoperative day 9. The patient initially completed five years of face-to-face, six-monthly reviews at the same tertiary cancer center, was then lost to follow-up for five years, and subsequently completed the 10-year follow-up through annual telehealth reviews.

Case 5

**Figure 6:**
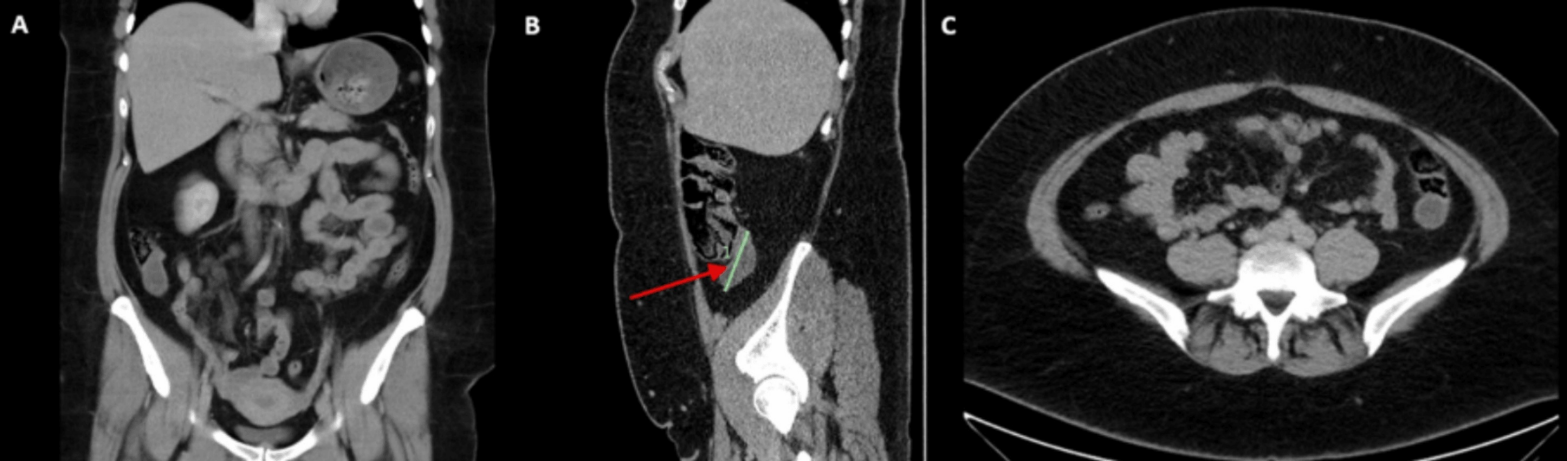
Computed tomography of the abdomen and pelvis with contrast (Case 5). (A) Coronal section; (B) sagittal section with the red arrow pointing to a tubular hypodense structure arising from the posterior cecum, posterior to the ileocecal junction and extending superiorly into the right flank, measuring approximately 6 cm in length and 2 cm in thickness; (C) sagittal section.

A 47-year-old female presented to an outpatient general surgery clinic with generalized abdominal pain. Her history included dyslipidemia, hypothyroidism, polycystic ovarian syndrome, and hyperandrogenism on metformin and spironolactone. On examination, she had RIF tenderness. A CTAP demonstrated a suspected AMN (Figure 6).

She underwent a laparoscopic appendicectomy, which revealed a dilated, mucinous appendiceal mass. Her postoperative course was uncomplicated, and she was discharged the following day. Histopathology revealed a non-ruptured LAMN with clear margins. She completed five years of disease-free surveillance locally via six-monthly reviews.

## Discussion

Rural perspective

This study is valuable in highlighting the challenges of managing AMNs rurally. Rural Australian centers report 7% of patients aged over 40 receiving appendicectomy were diagnosed with AMNs, which is higher than the overall incidence in prior literature [4]. We presented a series of five patients presenting to a rural center aged over 40 to educate them on early identification and treatment strategies for this rising condition.

First, our study emphasizes the importance of rural cancer MDTs. In our series, formal MDT review facilitated appropriate escalation of care in Cases 2 and 4 for CRS/HIPEC, which was vital as our center did not have access to these specialized therapies. MDT discussion also prompted the decision to operate in Case 1, with a view to reducing perforation risk via a safer, midline laparotomy approach. MDT access, however, is not universal across rural settings [5,6]. The reasons for this disparity are multifaceted, including remote geography, being less likely to have comprehensive patient information available [7], and less specialist input [6]. We combated these disparities by the use of videoconferencing [8] and hub-and-spoke models [9].

Second, this series also highlights the impact of distance on post-treatment care [5]. For LAMNs without perforation, acellular mucin, or extra-appendiceal spread we support evidence suggesting annual CT/magnetic resonance imaging (MRI) for five years, paired with CEA/CA 19-9/CA 125 every 6-12 months [10] - this low-risk pathology was observed in Cases 1, 3, and 5, and as they had their operation locally, these patients were followed-up close to home. For high-risk pathology, however, studies recommend more extensive follow-up [11], which may increase the patient’s travel burden. Contemporary series recommend LAMNs exhibiting perforation, cellular mucin, or extra-appendiceal mucin receive six-monthly CT/MRI for two years, then annually up to 10 years, with tumor markers at identical intervals [11]. This was pertinent for Case 4, who had a ruptured AMN and PMP, requiring transfer to a tertiary center for CRS/HIPEC. As he received specialized care at a metropolitan site, the patient was obligated to travel long distances for his appointments, and after completing five years of face-to-face reviews, he was lost to follow-up for five years. Fortunately, he was re-referred by his local general practitioner (GP), and completed his follow-up in 2025; this was assisted by the use of telehealth and local CT scans to reduce travel burden. Again, this underscores the geographical barriers that increase the risk of loss to follow-up [5], but also the strategies that can be used to mitigate this risk, including telehealth, communication with GPs, and the use of local radiology services.

Case 2 was initially referred to a tertiary center, but after being deemed a non-operative candidate, he was referred back to local oncology services for palliative chemotherapy. Prioritizing local end-of-life therapy in collaboration with tertiary specialists improves symptom control, reduces hospitalizations, and increases patient/caregiver satisfaction [12].

Recommendations on pathology, imaging, and surgical treatment

Our series supports evidence that inflammatory markers are non-specific for AMNs [2]. Case 1, for example, had normal inflammatory markers. When comparing the patients with ruptured appendices, Case 2 had markedly elevated WCC/CRP, while Case 4 only had mildly increased inflammatory markers. These results further substantiate that inflammatory markers lack diagnostic specificity for AMNs, even when ruptured [2]. AMNs may demonstrate elevated CEA/CA 19-9/CA 125 levels [2,13]; where a neoplastic process is suspected, tumor markers should be considered. Again, however, these tests lack specificity [2,13] and should be interpreted in conjunction with clinical and radiological findings.

CT is the gold standard for the preoperative diagnosis of AMNs [2]. The reported sensitivity and specificity are 83% and 92%, respectively [14], with high specificity for features such as cystic dilatation, mural calcification, and well-circumscribed low-attenuation lesions [2]. CT also has utility in differentiating AMNs from other cystic lesions [2] and in assessing the extent of peritoneal disease [2]. All our patients underwent preoperative CT, which enabled AMN identification, operative planning (Case 1), and detection of complications (rupture in Cases 2 and 4). We therefore recommend CT as the first-line imaging modality for AMN diagnosis and operative planning.

Surgical management of LAMNs depends on whether the disease is confined to the appendix or associated with extra-appendiceal spread. LAMN confined to the appendix is readily cured by simple appendicectomy, assuming negative margins and no evidence of perforation [15]. This was sufficient management for three patients (Cases 1, 3, and 5). Case 1 is important in demonstrating that, for large masses of unknown etiology, a midline laparotomy may be the safest approach to reduce the risk of rupture compared with a laparoscopic approach [16].

For ruptured LAMN, management is determined by whether the extra-appendiceal mucin is acellular (without viable epithelial cells) or cellular (containing neoplastic cells). If acellular mucin is present without peritoneal dissemination, an appendicectomy is sufficient [17]. Conversely, the presence of extra-appendiceal cellular mucin increases the risk of PMP and necessitates CRS/HIPEC [18], as observed in Case 4 due to the presence of mucinous neoplastic cells at the resection margin.

The need for right hemicolectomy has also been explored. The American Society of Colon and Rectal Surgeons (ASCRS) guidelines recommend right hemicolectomy only for patients with invasive adenocarcinoma with adverse features (i.e., high grade, lymphovascular invasion, mesoappendiceal or base involvement, tumor >2 cm, or positive epithelial margins) [19]. This recommendation is supported by other consensus guidelines [2,20]. This high-risk pathology was observed in Case 4, who underwent right hemicolectomy due to positive margins involving mucinous neoplastic cells.

Future directions

PREDICTORG (Unpublished clinical trial: Heriot A, Jain A. PREDICTORG: A Multicenter Personalised Medicine Approach in the Treatment of Colorectal Peritoneal Metastases; October 9, 2024) is a proposed multicenter trial that aims to integrate molecular, genomic, and histopathologic tumor samples profiling obtained at the time of CRS/HIPEC. By correlating these signatures with treatment response, PREDICTORG seeks to create a framework that can identify which patients will benefit from CRS/HIPEC and/or systemic therapy, thereby reducing unnecessary operations and individualizing treatment pathways for patients with peritoneal malignancy. Access to this new intervention will be essential for patient outcomes, and barriers of communication and distance for patients in rural settings will have to be addressed.

## Conclusions

Overall, this study is valuable in providing a rural perspective on AMN management. Due to the rising incidence, rural general surgeons will likely have to contend with this pathology and navigate changing management algorithms that may range from simple appendicectomy to specialized oncological management requiring tertiary center referral. In this study, we have given recommendations on pathological tests and imaging for preoperative identification, state-of-the-art surgical treatment, and strategies to combat resource limitations in rural centers - the importance of the rural cancer MDT and considering the impact of distance for rural patients attending postoperative appointments is particularly pertinent. Familiarity with these management principles is crucial for the rural general surgeon, especially for a rising and potentially deadly disease. 
